# Metal-to-Insulating Transition in the Perovskite System YSr_2_Cu_2_FeO_8−δ_ (0 < δ < 1) Modeled by DFT Methods

**DOI:** 10.1021/acs.inorgchem.2c03475

**Published:** 2023-02-14

**Authors:** Marianela Gómez-Toledo, Sara A. López-Paz, Susana García-Martín, M. Elena Arroyo-de Dompablo

**Affiliations:** † Departamento de Química Inorgánica, Universidad Complutense de Madrid, 28040 Madrid, Spain; ‡ Department of Quantum Matter Physics, University of Geneva, CH-1211 Geneva, Switzerland

## Abstract

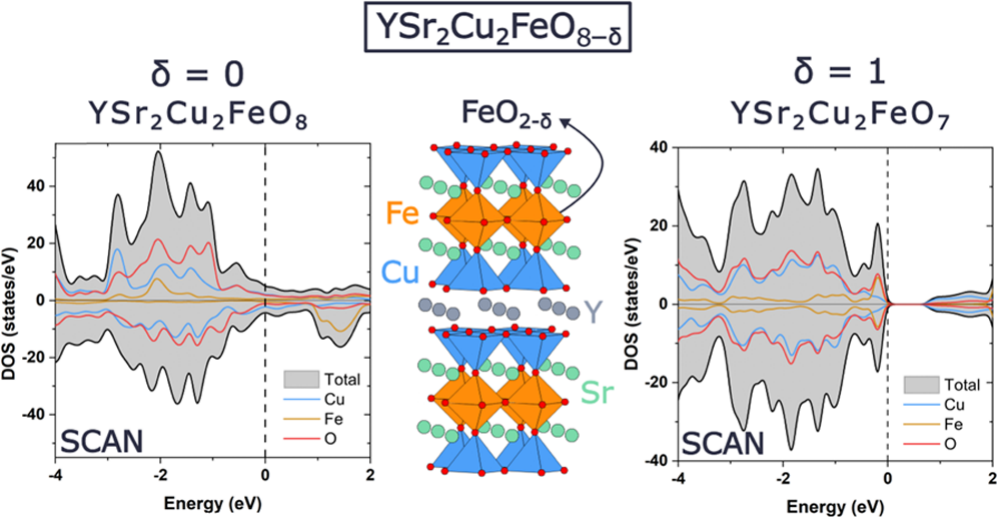

Progress in the design of functional perovskite oxides relies on advances in density functional theory (DFT) methods to efficiently and effectively model complex systems composed of several transition-metal ions. This work reports the application of DFT methods to investigate the electronic structure of the YSr_2_Cu_2_FeO_8−δ_ (0 < δ < 1) family in which the insulating, metal, or superconducting behaviors and even anion conductivity can be tuned by modifying the oxygen content. In particular, we assess the performance of the generalized gradient approximation (GGA), its Hubbard-*U* correction (GGA + *U*), and the strongly constrained and appropriately normed (SCAN) to model the metallic (idealized YSr_2_Cu_2_FeO_8_) and insulating (idealized YSr_2_Cu_2_FeO_7_) phases of the system. The analysis of the DFT results is supported by DC resistivity measurements that denote the metal character of the synthesized YSr_2_Cu_2_FeO_7.86_ and the semiconducting character of YSr_2_Cu_2_FeO_7.08_ prepared under reducing conditions. In addition, the band gap of YSr_2_Cu_2_FeO_7.08_, in the range of 0.73–1.2 eV, has been extracted from diffuse reflectance spectroscopy (DRS). While the three methodologies (GGA, GGA + *U*, SCAN) permit the reproduction of the crystal structures of the synthetized oxides (determined here in the case of YSr_2_Cu_2_FeO_7.08_ by neutron powder diffraction (NPD)), the SCAN emerges as the only one capable to predict the basic electronic and magnetic properties across the YSr_2_Cu_2_FeO_8−δ_ (0 < δ < 1) series. The picture that emerges for the metal (δ = 0) to insulating (δ = 1) transition is the one in which oxygen vacancies contribute electrons to the filling of the Cu/Fe-3*d_x^2^–y__^2^_* states of the conduction band. These results validate the SCAN functional for future DFT investigations of complex functional oxides that combine several transition metals.

## Abstract 2

The performance of DFT methods in the investigation of complex transition-metal oxides is evaluated taking as a case study the YSr_2_Cu_2_FeO_8−δ_ (0 < δ < 1) system. The SCAN functional properly accounts for the structural, electronic, and magnetic features of the synthesized YSr_2_Cu_2_FeO_7.85_ and YSr_2_Cu_2_FeO_7.08_ terms. While the SCAN functional successfully models the metal-to-insulating transition in YSr_2_Cu_2_FeO_8−δ_, the GGA (PBE) and GGA + *U* (PBE + *U*) methods offer a poor description of this system.

## 1 Introduction

Perovskite-type oxides (ABO_3_ and related materials) exhibit a broad range of functional properties that derive in multiple and relevant applications.^1^ To better understand the properties and optimize the applications of these oxides, a profound knowledge of their crystal and electronic-structure relationships is compulsory. Nowadays, electronic-structure calculations based on density functional theory (DFT) are extensively applied to successfully predict and design materials with particular properties. Since the development of DFT by Holenberg, Kohn, and Sham,^2,3^ a great deal of improvement on exchange-correlation (XC) functionals has been reported, such as the widely used generalized gradient approximation (GGA) developed in the late 1980s.^4,5^ The GGA functionals have offered some acceptable results in the modeling of perovskite oxides based on *d*^0^ transition metals (TM).^6−9^ However, the overestimation of electron delocalization and metallic character is a known failure of DFT methods for systems with localized and strongly interacting *d*-electrons and *f*-electrons.^10−13^ In the 1990s, the DFT + *U* method introduced an explicit treatment of electron correlation with a Hubbard-like model for a subset of states in the system.^10,14^ Vast literature demonstrates the suitability of the DFT + *U* approach to investigate TM oxides with strong Coulomb correlations. More recently, the strongly constrained and appropriately normed (SCAN) meta-GGA functional^15^ has shown good performance to model the basic physical properties and phenomena associated to correlated oxides.^16,17^ For instance, investigations on perovskite-related oxides using meta-GGA functionals (and particularly SCAN) have accurately described the transition from the insulating to the metallic character of La_2_CuO_4_ by substitution of La by Sr in the superconducting system;^18^ the layered ordering stabilization of the Bi_2_MFeO_6_ perovskites (M = Al, Ga, In) induced by the Fe–magnetic interactions;^19^ the effect of the hole and electron doping in the electronic structure of Sm_1–*x*_M*_x_*NiO_3_ (M = Ca, Ce);^20^ and have predicted the structural cubic-symmetry breaking, band-gap existence, and magnetic behavior in ABO_3_ perovskites with B = 3*d*-TM.^21^

**Figure 1:**
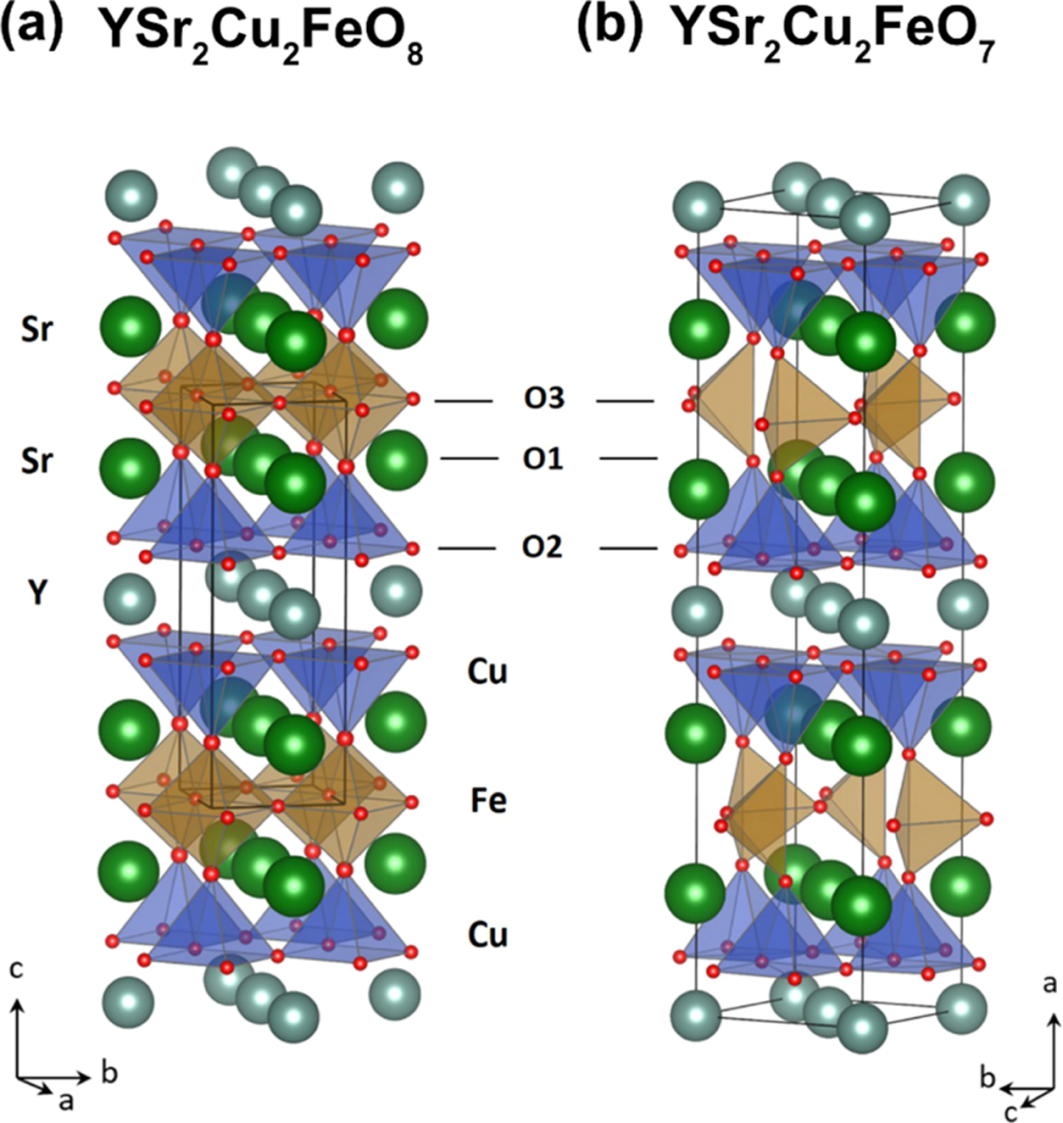
Crystal structure representations of (a) idealized stoichiometric oxide YSr_2_Cu_2_FeO_8_ (*a*_p_ × *a*_p_ × 3*a*_p_ unit cell, S.G. *P*4/*mmm*) and (b) idealized stoichiometric oxide YSr_2_Cu_2_FeO_7_ (6*a*_p_ × √2*a*_p_ × √2*a*_p_ unit cell, S.G. *Ima*2).

Despite the substantial progress in the computational investigation of perovskites, the scenario complicates when different TM ions occupy the B positions, even if this occurs in an ordered manner. Hence, assessing the performance of DFT methodologies and, specifically, of the recently developed SCAN functional at a basic property-level prediction—crystal, electronic, and magnetic structures—is a prerequisite to further investigating the particular properties of complex perovskite oxides. In the present work, we focus on the YSr_2_Cu_2_FeO_8−δ_ (0 < δ < 1) family derived from the so-called “YBaCuO” superconducting cuprates.^22,23^Figure 1a,b displays the graphic representation of the crystal structure of the idealized stoichiometric endmembers YSr_2_Cu_2_FeO_8_ and YSr_2_Cu_2_FeO_7_ (δ = 0 and 1, respectively). The structures consist of an alternation of FeO_2−δ_/SrO/CuO_2_/Y/CuO_2_/SrO layers along the *c*-axis or *a*-axis (space group (S.G.) *P*4/*mmm* and S.G. *Ima*2 settings, respectively). The idealized YSr_2_Cu_2_FeO_8_, with [FeO_6_] octahedral units in the Fe layers, presents a tetragonal (S.G. *P*4/*mmm*) crystal structure with *a*_p_ × *a*_p_ × 3*a*_p_ unit cell dimensions (*a*_p_ refers to the lattice parameter of the cubic perovskite structure).^24^ Lowering the oxygen content (δ > 0) creates vacancies in the O3 positions of the FeO_2−δ_ layer (Figure 1) so that in the YSr_2_Cu_2_FeO_8−δ_ family, the Fe atoms may adopt octahedral, tetrahedral, and/or square pyramid coordination.^23−27^ In combination with the Y and Sr ordering, there is layered ordering of the coordination polyhedra (octahedra O, square pyramid SP, and tetrahedra T) around the Fe and Cu atoms in the manner O/SP/SP/O in the idealized YSr_2_Cu_2_FeO_8_ and like T/SP/SP/T in the idealized YSr_2_Cu_2_FeO_7_. In addition, in the idealized YSr_2_Cu_2_FeO_7_, the different orientation of the tetrahedral coordination polyhedra of the Fe atoms leads to a sixfold superstructure (6*a*_p_ × √2*a*_p_ × √2*a*_p_ within the *Ima*2 space group).^23,28^

YSr_2_Cu_2_FeO_8−δ_ (0 < δ < 1) compounds show a nonstoichiometric anion sublattice resulting from the interplay between different oxidation states of Fe and Cu atoms.^22,23,29^ Oxides of the system, with different oxygen content and properties, have already been reported. The highly oxidized term YSr_2_Cu_2_FeO_7.85_ obtained experimentally in ozone flow, with Fe in the unusual 4+ formal oxidation state and mixed Cu^3+^ and Cu^2+^ valences, is a metallic oxide with a superconducting transition at *T*_c_ = 70 K.^24^ The compound presents magnetic ordering arising from the ferromagnetic (FR) interactions below *T*_N_ = 110 K between Fe^4+^ cations with parallel in-plane spins in the FeO_2−δ_ layers coupled with a soft character of the antiferromagnetic (AFM) interactions between layers.^24^ The low oxygen content terms YSr_2_Cu_2_FeO_7.08_,^27^ YSr_2_Cu_2_FeO_7.11_,^28^ and YSr_2_Cu_2_FeO_7.04_,^26^ prepared under reducing conditions, are insulators with mainly Fe^3+^ and Cu^2+^. In between those extremes, air-synthesized YSr_2_Cu_2_FeO_7.56_, mixed valence Fe^3+^/Fe^4+^ and Cu^2+^/Cu^3+^ oxide, exhibits an interesting electrochemical behavior associated with catalytic activity in the oxygen reduction reaction (ORR), being a potential air electrode for solid oxide fuel cells (SOFCs).^26,27^

Modeling the evolution of the electronic structure of YSr_2_Cu_2_FeO_8−δ_ from the metallic phase (δ ∼ 0) to the insulating one (δ ∼ 1), which is to say, the metal-to-insulating transition as a function of the oxygen content, constitutes a challenge for DFT methods, apart from the relevance of tuning the electronic properties of these materials to manage particular applications. Indeed, few computational works deal with the two facts that are found in YSr_2_Cu_2_FeO_8−δ_ oxides: first, the combination of two transition-metal ions in the B positions of the crystal structure, and second, the occurrence of oxygen nonstoichiometry. Both aspects highly condition the electronic properties of the materials.

We here report DFT calculations for the idealized stoichiometric compounds YSr_2_Cu_2_FeO_8_ and YSr_2_Cu_2_FeO_7_ as representatives of the experimentally obtained YSr_2_Cu_2_FeO_7.85_ and YSr_2_Cu_2_FeO_7.08_. The calculations have been performed within the GGA-Perdew, Burke, and Ernzerhof (PBE) functional (hereafter denoted as simply GGA), its Hubbard correction PBE + *U* (denoted as GGA + *U*), and the meta-GGA-SCAN functional (denoted as SCAN). We demonstrate that the three methodologies fairly reproduce the crystal structures. However, the prediction of the electrical and magnetic properties depends on the functional used for the calculation. While the metallic behavior and magnetic features of the YSr_2_Cu_2_FeO_8_ phase are well described with the GGA and SCAN functionals, the SCAN and GGA + *U* offer the best approaches to investigate the YSr_2_Cu_2_FeO_7_ compound. We also support the electronic-structure calculations with resistivity experiments that confirm the metal-to-insulating transition in the YSr_2_Cu_2_FeO_8−δ_ (0 < δ < 1) system. In addition, the optical band gap of YSr_2_Cu_2_FeO_7.08_ has been determined from diffuse reflectance spectroscopy (DRS).

## 2 Methodology

### 2.1 Experimental Section

Our previous works cover the synthesis and structural characterization of several YSr_2_Cu_2_FeO_8−δ_ oxides.^24,27,30^ In this work, polycrystalline YSr_2_Cu_2_FeO_8−δ_ (δ = 0.15 and 0.92) compounds have been prepared by the conventional ceramic method in air. The intimate mixtures of Fe_2_O_3_ (Aldrich 99.99%), CuO (Aldrich 99.9999%), previously decarbonated Y_2_O_3_ (Aldrich 99.9%), and previously dehydrated SrCO_3_ (Aldrich 99.9%) in the stoichiometric relations were subjected to an initial treatment at 1173 K for 12 h in air. The resulting powders were ground in an agate mortar, pelletized, and subjected to several treatments at 1253 K in air, for 72 h, with intermediate grindings. The air-prepared sample was heated at 1023 K for 24 h in N_2_ flow to obtain YSr_2_Cu_2_FeO_7.08_ oxide and the N_2_ prepared compound was oxidized in ozone at 473 K to obtain YSr_2_Cu_2_FeO_7.85_.

The crystal structure of the YSr_2_Cu_2_FeO_7.08_ compound at room temperature (RT) was determined by neutron powder diffraction (NPD) using the high-resolution D2B instrument at the ILL (Grenoble, France) with a wavelength of λ = 1.594 Å. Rietveld refinements were performed following the Fullprof software.^31^

DC electrical resistivity measurements of sintered pellets of YSr_2_Cu_2_FeO_7.85_ and YSr_2_Cu_2_FeO_7.08_ have been performed in the temperature region 5 < *T* < 300 K using a Quantum Design PPMS device. Four-probe electrical contacts were made using conductive mixed silver paste and gold wires. The resistivity was measured in sweep mode with a heating rate of 2 K/min.

DRS measurements have been carried out to determine the optical band gap in YSr_2_Cu_2_FeO_7.08_. The experiments have been performed in a Cary 5G spectrophotometer with an external integrating sphere. The measurement range was 300–3300 nm and the data interval was 1 nm. As stated by Kubelka and Munk,^32^ diffuse reflectance spectra can be transformed into absorption spectra through the Kubelka–Munk function (*F*(*R*_∞_), eq 1)
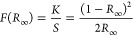
1where *R*_∞_ is the absolute reflectance of an infinitely thick specimen and *K* and *S* are the absorption and scattering coefficients, respectively. *F*(*R*_∞_) is proportional to the extinction coefficient (α), which can be expressed by eq 2, according to the Tauc method^33^
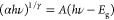
2where *h* is the Plank constant, ν is the photon frequency, *E*_g_ is the band-gap energy, and *A* is a constant. The γ factor considers the nature of the electronic transition, being equal to 1/2 for allowed direct and 2 for allowed indirect transition.

Replacing α with *F*(*R*_∞_) results in the following expression (eq 3)
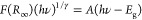
3The band gap can thus be extracted from diffuse reflectance spectra transformed according to eq 3. Linear fitting of the Tauc plot followed by extrapolating to the *x*-axis intersection gives the band-gap value.

### 2.2 Computational Section

Calculations for the idealized stoichiometric endmembers of the YSr_2_Cu_2_FeO_8−δ_ family, YSr_2_Cu_2_FeO_8_ and YSr_2_Cu_2_FeO_7_, have been performed using the ab initio total-energy and molecular dynamics program Vienna *ab initio* simulation package (VASP) developed at the Universität Wien.^34,35^ The interaction of core electrons with the nuclei is described by the projector augmented wave (PAW) method^36^ with 2*s*^2^2*p*^4^ of O, 3*s*^2^3*p*^6^3*d*^7^4*s*^1^ of Fe, 3*p*^6^3*d*^10^4*p*^1^ of Cu, 4*s*^2^4*p*^6^5*s*^2^ of Sr, and 4*s*^2^4*p*^6^4*d*^1^5*s*^2^ of Y treated as valence electrons. For the GGA approximation, we selected the exchange and correlation functional form developed by Perdew, Burke, and Ernzerhof (PBE).^37^ On the other hand, for the meta-GGA approximation, the strongly constrained and appropriately normed (SCAN)^15^ functional was used. In all cases, the energy cut off for the plane wave basis set was kept fixed at a constant value of 600 eV throughout the calculations. The integration in the Brillouin zone is done on appropriate sets of *k*-points determined by the Monkhorst–Pack scheme. The *k*-point meshes were set at 2 × 8 × 8 for YSr_2_Cu_2_FeO_7_ and 10 × 10 × 4 for YSr_2_Cu_2_FeO_8_, using a Gaussian smearing parameter of 0.05 eV. For the density of states (DOS) calculations, the tetrahedron method with Blöchl corrections^38^ was used. Self-consistency was achieved with a tolerance in total energy of 1 × 10^–4^ eV for geometry optimization and 1 × 10^–6^ eV for DOS calculations.

A local Hubbard-*U* (GGA + *U*) was added to Fe and Cu atoms following the simplified rotationally invariant framework developed by Dudarev et al.^39^ Typically, *U* is formulated as *U*_eff_ = *U* – *J*, where *U* is the onsite Coulomb term and *J* the exchange term. In this work, this effective parameter is simply referred to as *U*. The *J* value was fixed to 1 eV. An effective *U* value of 4 eV was used for the *d* orbitals of Cu and Fe, that were found appropriate in previous GGA + *U* investigations.^13,40−42^ The local magnetic moments are taken from the difference between the projected electron density of up and down spins onto 1 Å radius sphere. Bader charge analysis^43^ was performed on the charge density files^44^ using the pymatgen package.^45^

In the present work, we have performed calculations using the idealized stoichiometric compositions YSr_2_Cu_2_FeO_7_ and YSr_2_Cu_2_FeO_8_ and perfect Cu/Fe ordering within the crystal structures. The crystallographic model of YSr_2_Cu_2_FeO_8_ oxide has been constructed taking the initial positions from YSr_2_Cu_2_FeO_7.86_ (ICSD file 11514),^24^ considering a ferromagnetic 2 × 2 × 1 superstructure of the tetragonal *a*_p_ × *a*_p_ × 3*a*_p_ unit cell shown in Figure 1a (that is, Y_4_Sr_8_Cu_8_Fe_4_O_32_ composition). For YSr_2_Cu_2_FeO_7_ oxide, the 6*a*_p_ × √2*a*_p_ × √2*a*_p_ (S.G. *Ima*2) unit cell has been used for the calculations (Y_4_Sr_8_Cu_8_Fe_4_O_28_), corresponding to an ideal arrangement of the tetrahedral chains (Figure 1b). The initial cell parameters and atomic positions of YSr_2_Cu_2_FeO_7_ were taken from the ICSD record of isostructural YSr_2_Cu_2_GaO_7_.^46^ Although the magnetic structure of YSr_2_Cu_2_FeO_8−δ_ phases with high δ values (δ ∼ 1) has not yet been experimentally determined, magnetic interactions associated to Fe^3+^ and Cu^2+^ cations are expected, and, as explained below, different magnetic structures have been considered.

## 3 Results and Discussion

### 3.1 Experimental Section

**Figure 2:**
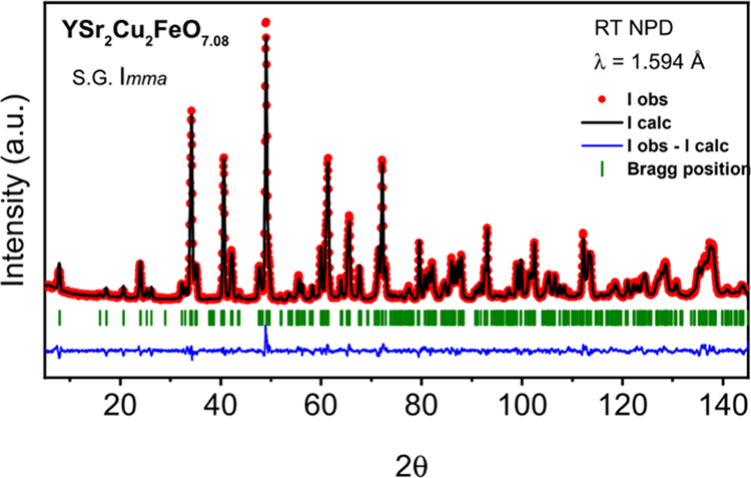
Rietveld refinement of the room temperature (RT) NPD pattern of YSr_2_Cu_2_FeO_7.08_.

The crystal structure and superconducting behavior of the compound with the highest oxygen content, YSr_2_Cu_2_FeO_7.86_, has previously been reported.^24^ In this work, the crystal structure of YSr_2_Cu_2_FeO_7.08_ is refined by NPD. Table 1 lists the results of the Rietveld refinement of the NPD pattern of YSr_2_Cu_2_FeO_7.08_ (Figure 2). The refined occupation factors of 8i and 8h crystallographic positions reveal certain disorder (antisite location) of the Fe and Cu atoms. Note that consistently with the oxygen content (δ = 0.92 in YSr_2_Cu_2_FeO_8−δ_), there is an occupation of 0.574 for O3 in 8i positions. In the idealized term δ = 1 (YSr_2_Cu_2_FeO_7_) where only tetrahedral-Fe is present, the fractional occupancy of O3 is of 0.5. Importantly, the results indicate that corrugation of the [FeO_4_] tetrahedral units and their relative orientation along the stacking direction originate an additional superstructure with a diagonal orthorhombic unit cell of dimensions √2*a*_p_ × 6*a*_p_ × √2*a*_p_ in the *Imma* space group (note the change of S.G. setting as described in Table 2). Therefore, the refined crystal structure of YSr_2_Cu_2_FeO_7.08_ is in close agreement with the one reported by Mochiku et al. (ICSD file 151653) for the YSr_2_Cu_2_FeO_7.11_ compound.^28^ The crystallographic model with the *Imma* space group accounts for the presence of two types of tetrahedral chains in the Fe layers, which are left- and right-hand rotated. In this sense, the crystal structure of YSr_2_Cu_2_FeO_7.08_ can be considered as a disordered version of the model crystal structure of the stoichiometric YSr_2_Cu_2_GaO_7_ determined by Roth et al. (ICSD file 71263, used for the DFT calculations)^46^ in which the presence of a single chain orientation is well captured within the *Ima*2 space group, as it is represented in Figure 1b.

**Table 1:** Atomic Positions and Cell Parameters Obtained from the Rietveld Refinement of the NPD Data for YSr_2_Cu_2_FeO_7.08_^a^

atom	site	*x*	*y*	*z*	Biso (Å)	occ.
Y	4a	0	0	0	0.20(4)	1
Sr	8h	0	0.34911(7)	0.0067(8)	0.68(4)	1
Fe1/Cu1	8i	0.0440(8)	0.25	0.5584(5)	1.0(7)	0.426(7)/0.074(7)
Cu2/Fe2	8h	0	0.42610(8)	0.5000(5)	0.28(3)	0.85(1)/0.15(1)
O1	8h	0	0.3257(1)	0.4739(7)	1.30(5)	1
O2a	8g	0.25	0.0630(1)	0.25	0.39(2)	1
O2b	8g	0.25	–0.0649(1)	0.25	0.39(2)	1
O3	8i	0.390(1)	0.25	0.616(1)	2.1(1)	0.574(3)

a S.G. *Imma* (no. 74); *a* = 5.4053(1) Å, *b* = 22.9136(5) Å, *c* = 5.4577(1) Å, *V* = 675.98(2) Å^3^, δ = 0.92; *R*_p_ = 3.62%; *R*_wp_ = 4.66%; χ^2^ = 4.22.

**Table 2:** Calculated Unit Cell Parameters (Å), Unit Cell Volume (Å^3^), Volume per Atom (Å^3^), and Selected Bond Lengths (Å) for YSr_2_Cu_2_FeO_7_ and YSr_2_Cu_2_FeO_8_ Idealized Oxides^a^

YSr_2_FeCu_2_O_8_	experimental YSr_2_FeCu_2_O_7.85_	GGA	GGA + *U*	SCAN	YSr_2_FeCu_2_O_7_	experimental^b^ YSr_2_FeCu_2_O_7.08_	C-AF GGA	C-AF GGA + *U*	C-AF SCAN
*a*, *b*	3.8145(3)	3.8252	3.8315	3.7861	*a*	22.9136(5)^b^	23.1337	23.1424	22.8529
*c*	11.327(7)	11.3803	11.5457	11.2755	*b*	5.4577(1)^b^	5.5007	5.5069	5.4411
*V*	164.81(1)	166.52	169.50	161.62	*c*	5.4053(1)^b^	5.4241	5.4265	5.3744
*V* per atom	11.77	11.89	12.10	11.54	*V*	675.98(2)	690.13	691.63	668.22
*d* Fe–O3	1.9072(2)	1.9126	1.9157	1.8930	*V* per atom	13.00	13.27	13.30	12.85
*d* Fe–O1	1.843(4)	1.8550	1.8813	1.8417	mean *d* Fe–O	1.8732	1.8754	1.9014	1.8732
*d* Fe–O1/d Fe–O3	0.9663	0.9699	0.9820	0.9729	Fe–O3–Fe	124.6°	126.0°	122.9°	124.5°
*d* Cu–O2	1.9244(4)	1.9355	1.9473	1.9147	*d* Cu–O2	1.933(2)/1.934(2)	1.9365/1.9456	1.9413/1.9507	1.9267/1.9338
*d* Cu–O1	2.117(4)	2.1134	2.1197	2.0812	*d* Cu–O1	2.302(4)	2.4327	2.3883	2.3449
*d* Cu–O1/*d* Cu–O2	1.1000	1.0919	1.0885	1.0870	*d* Cu–O1/*d* Cu–O2	1.1906	1.2533	1.2273	1.2148
*d* Cu–Cu	3.407(4)	3.4435	3.5439	3.4297	*d* Cu–Cu	3.384(4)	3.2837	3.3506	3.3095
*d* Cu–Fe	3.960(3)	3.9684	4.0009	3.9229	*d* Cu–Fe	4.056(2)	4.1639	4.1337	4.0815

a The experimental data of YSr_2_Cu_2_FeO_7.86_ (ICSD—11514) and YSr_2_Cu_2_FeO_7.08_ (this work) are included for comparison. For the GGA + *U* method, a value of *U* = 4 eV is used for both Cu and Fe.

b Lattice parameters given in the setting for the S.G. *Ima*2. The unit cell metrics corresponding with the S.G. *Imma* (Table 1) is *a* (*Imma*) < > *c* (*Ima*2); *b* (*Imma*) < > *a* (*Ima*2); and *c* (*Imma*) < > *b* (*Ima*2).

**Figure 3:**
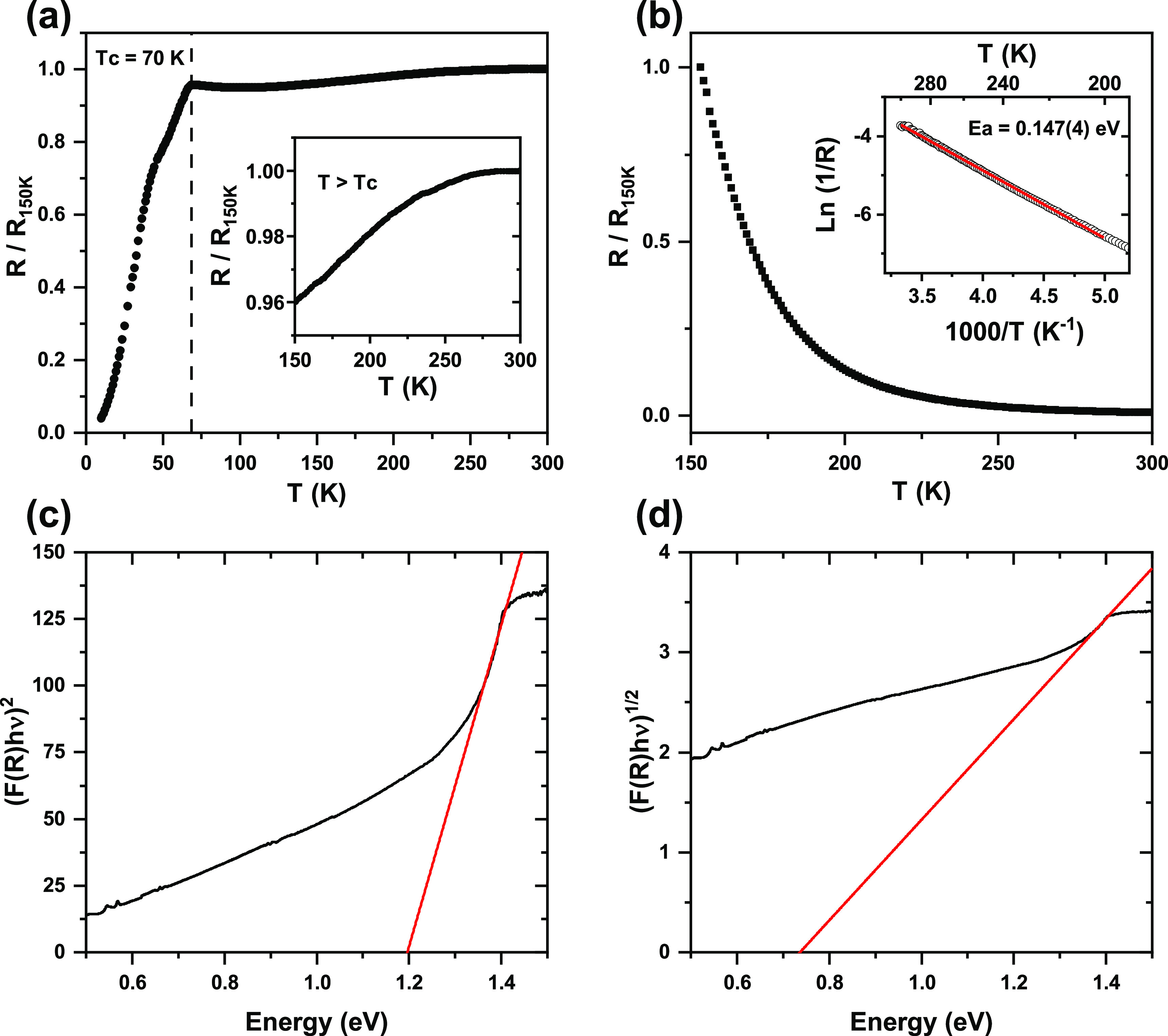
Normalized DC resistance for (a) YSr_2_Cu_2_FeO_7.85_ and (b) YSr_2_Cu_2_FeO_7.08_ oxides. The Arrhenius plot used for the determination of the activation energy (*E*_a_) is also shown in panel (b) as an inset. Tauc plot for YSr_2_Cu_2_FeO_7.08_ oxide considering direct (c) and indirect (d) allowed electronic transitions.

The variation of the resistivity with the temperature of YSr_2_Cu_2_FeO_7.86_ shows the expected superconducting/metallic transition at *T*_c_ = 70 K (Figure 3a).^24^ Above the *T*_c_, the increase of the electrical resistance with temperature reflects the metallic character (inset in Figure 3a). On the contrary, YSr_2_Cu_2_FeO_7.08_ oxide presents a semiconducting/insulating transition near 200 K (Figure 3b). The logarithmic plot of resistance (inset in Figure 3b) shows an activated behavior in the 200–300 K temperature range, with an activation energy (*E*_a_) of 0.147(4) eV.

The optical band gap of the YSr_2_Cu_2_FeO_7.08_ phase has been extracted from DRS measurements. The Tauc plot and Kubelka–Munk analysis offer an approximate estimation of optical band gap.^32,33,47^ However, if the nature of the electronic transition is not well known (as in the present case), the γ parameter (eq 2) value becomes an important source of uncertainty. For YSr_2_Cu_2_FeO_7.08_, considering a direct allowed electronic transition, from the Tauc plot we derive a band-gap value of 1.2 eV, while a band gap of 0.73 eV results if an indirect electronic transition is considered (Figure 3c). Methods such as photoluminescence (PL)^48^ and X-ray photoelectron spectroscopy (XPS)^49^ that offer an accurate band-gap determination are out of the scope of this paper. Given the above, this work considers the interval 1.2–0.73 eV to compare the band gap of YSr_2_Cu_2_FeO_7.08_ with the DFT-calculated band gap.

### 3.2 DFT Investigation of YSr_2_Cu_2_FeO_8−δ_

#### 3.2.1 Metallic YSr_2_Cu_2_FeO_8_ Phase

Regardless of the constraints in comparing the experimental data of YSr_2_Cu_2_FeO_7.85_ (δ = 0.15) and the idealized model YSr_2_Cu_2_FeO_8_ (δ = 0), all of the utilized DFT methodologies reproduce the experimental lattice parameters and bond distances (Table 2), with small deviations ranging from a maximum of 4% (GGA + *U*) to a minimum of 0.2% (SCAN). The compression of the Fe octahedra (*d* Fe–O1/*d* Fe–O3 < 1) and the elongation of the Cu-square pyramid (*d* Cu–O1/*d* Cu–O2 > 1) along the *c*-axis are also well captured. There are, however, some subtle differences in the predicting capabilities of the four DFT methodologies. Both the GGA and GGA + *U* methods tend to produce larger lattice parameters, bonding distances (see for instance Fe–O1), and cell volume than the SCAN functional. For the YSr_2_Cu_2_FeO_8−δ_ family, both *a* and *c* lattice parameters decrease with increasing oxygen content,^23^ and hence the idealized stoichiometric compound YSr_2_Cu_2_FeO_8_ should have a lower volume than the synthesized YSr_2_Cu_2_FeO_7.85_. In this respect, the SCAN functional offers superior performance in predicting such volume reduction.

The calculated magnetic moments and effective Bader charges (Table 3) differ among the different methodologies due to the distinct description of the transition-metal–oxygen bonding. The effective charge of all ions increases from the GGA to the GGA + *U*, as expected, due to the decreasing covalency of the Fe–O and Cu–O chemical bonds when correlation effects are taken into account. The poor electron localization in the GGA and SCAN approximations yields low magnetic moments on the transition-metal ions (0–0.2 and 2.7–3.0 μ_B_ per Cu and Fe atom, respectively). Treating the correlation effects results in higher electron localization and larger calculated magnetic moments for Cu atoms (0.5 μ_B_) and Fe atoms (3.4 μ_B_). Neutron diffraction results and Mössbauer spectra of YSr_2_Cu_2_FeO_7.85_ yielded a magnetic moment μ_0_ (Fe) = 1.7–2 μ_B_ per Fe atom that the authors attributed to Fe^4+^ ion in low-spin configuration (*t*_2g_^4^*e*_g_^0^).^24^ No magnetic moment was detected for Cu^3+/2+^ ions. In this regard, although quantitative comparison of magnetic moments with experiments is complicated due to the observed Cu/Fe antisite mixing^24,50^ and differences in the oxygen content, the GGA, and SCAN methodologies offer a more appropriate description than the GGA + *U*.

**Table 3:** Calculated Local Magnetic Moments μ (in μ_B_ per Atom) and Effective Bader Charges (*Q*) for Idealized YSr_2_Cu_2_FeO_7_ and YSr_2_Cu_2_FeO_8_ Oxides^a^

YSr_2_Cu_2_FeO_8_				C-AFM YSr_2_Cu_2_FeO_7_			
	GGA	GGA + *U*	SCAN		GGA	GGA + *U*	SCAN
*Q* (Fe)	1.796	1.881	1.930	*Q* (Fe)	1.481	1.643	1.628
*Q* (Cu)	1.073	1.090	1.157	*Q* (Cu)	0.942	1.010	1.081
*Q* (O1)	–1.158	–1.187	–1.228	*Q* (O1)	–1.267	–1.324	–1.347
*Q* (O2)	–1.187	–1.192	–1.232	*Q* (O2)	–1.235	–1.260	–1.310
*Q* (O3)	–1.125	–1.175	–1.175	*Q* (O3)	–1.210	–1.297	–1.300
μ (Fe)	2.7	3.4	3.0	μ (Fe)	3.6	4.1	3.9
μ (Cu)	0.0	0.5	0.2	μ (Cu)	0.0	0.5	0.5
μ (O1)	0.1	0.0	0.1	μ (O1)	0.2	0.2	0.2
μ (O2)	0.0	0.1	0.0	μ (O2)	0.0	0.0	0.0
μ (O3)	0.2	0.1	0.1	μ (O3)	0.0	0.0	0.0

a Within the GGA + *U* method, a value of *U* = 4 eV is used for both Cu and Fe.

**Figure 4:**
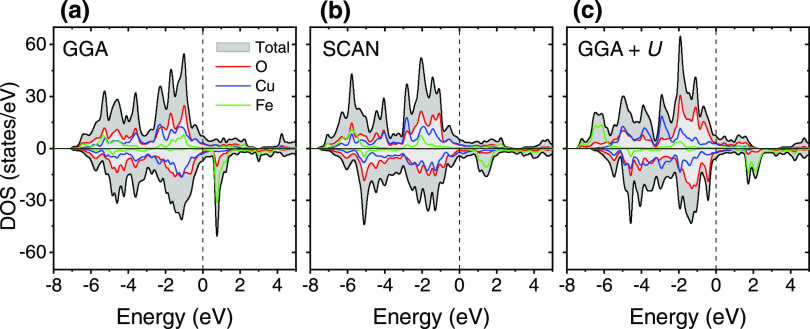
Calculated total and atom-projected density of states for YSr_2_Cu_2_FeO_8_: (a) GGA functional, (b) SCAN functional, and (c) GGA + *U* method. The Fermi level is set as the zero of energy. Up-spin (or majority) and down-spin (or minority) contributions are shown in the upper and lower parts of the panels, respectively. Color code: total DOS in gray, Cu contribution in blue, Fe contribution in green, and O contribution in red. DOS units refer to the calculated cell.

The three DFT approximations predict the metallic behavior of the YSr_2_Cu_2_FeO_8_ oxide (see calculated DOS in Figure 4). Compared to the GGA method, the introduction of the *U* term produces a downshift of the occupied Cu/Fe-3*d* states. The *U* parameter keeps the 3*d* orbitals of Fe ions atomic-like, diminishing their hybridization with the 2*p* orbitals of the oxygen ions (less covalent Fe–O bonding). Although the Fe down-spin states are partially occupied under all approximations, the occupancy is the lowest for the GGA + *U* methodology, therefore resulting in the larger magnetic moment of 3.4 μ_B_ per Fe atom, which is close to that expected for a high-spin Fe^4+^ configuration (*t*_2g_^3^*e*_g_^1^), and deviates from the experimental observations.

#### 3.2.2 Insulating YSr_2_Cu_2_FeO_7_ Phase

**Figure 5:**
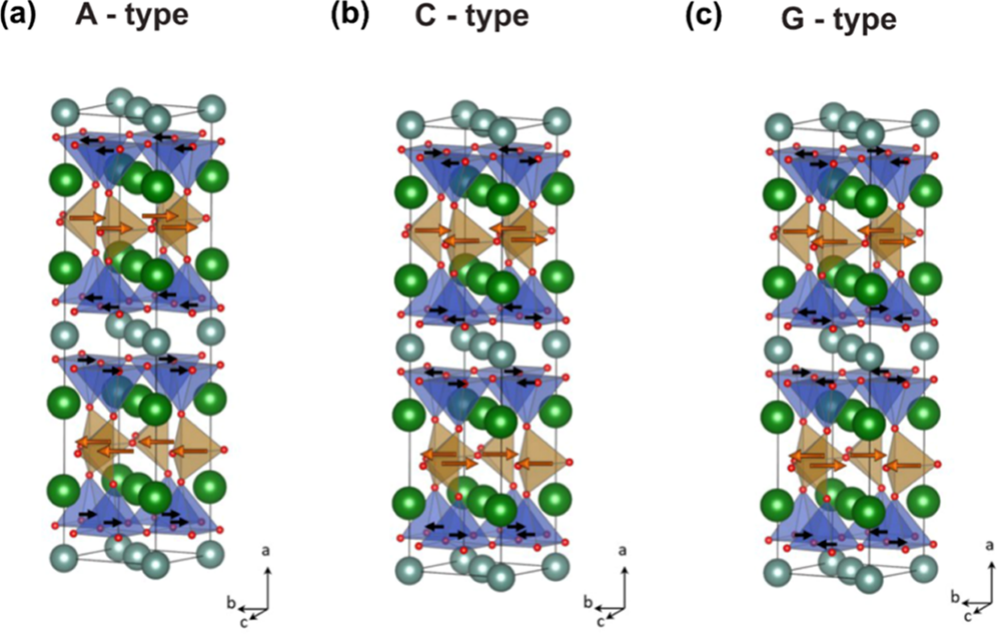
(a–c) Magnetic structures (C, G, A) used to simulate YSr_2_Cu_2_FeO_7_ (S.G. *Ima*2). Arrows indicate the spin orientation of the Fe and Cu atoms within the magnetic structure. Color code: Fe atoms in brown, Cu atoms in blue, Sr atoms in green, Y atoms in gray, and O atoms in red.

Figure 5a–c shows the magnetic structures considered to model the idealized YSr_2_Cu_2_FeO_7_ oxide. In the A-type structure, there are FM Cu/Fe in-plane interactions, while the planes order antiferromagnetically along the *a*-axis (tetragonal *c*-axis in YSr_2_Cu_2_FeO_8_). In the C-type, for each magnetic site, an in-plane AFM configuration between nearest-neighbor spins exits, while a FM configuration is set between the Fe and Cu planes. However, the sign reverses across the Y-layers due to the AFM direct exchange between Cu^2+^ cations at the face-confronted pyramids. Within each (CuO_2_–SrO–FeO–SrO–CuO_2_) block, a C-type magnetic ordering is thus defined since the Cu/Fe magnetic moments can be grouped as chains along the *a*-axis (tetragonal *c*-axis in YSr_2_Cu_2_FeO_8_). The G-type presents the same AFM in-plane magnetic ordering of the C-type, but with AFM interactions between the Cu and Fe planes.

The four magnetic states tested to model YSr_2_Cu_2_FeO_7_ follow the energetic ordering: FM ∼ A-AFM ≫ G-AFM ∼ C-AFM (Table 4). The large stabilization of the G and C-AFM types (about 0.4 eV/fu respective to the FM configuration) denotes strong in-plane Cu/Fe AFM interactions. On the other hand, the small energy difference between G and C-AFM types, and between A-AFM and FM types, (5 meV/fu) suggests weak interplane magnetic interactions. The GGA, GGA + *U*, and SCAN methodologies predict the C-AFM to be the ground state, which has therefore been chosen to investigate the crystal and electronic structures of YSr_2_Cu_2_FeO_7_.

**Table 4:** Calculated Total-Energy Differences for Magnetic Configurations (Figure 5a–c) in YSr_2_Cu_2_FeO_7_ (in eV/fu)^a^

type	GGA	GGA + *U*	SCAN
A-AFM	0.020	0.020	–0.075
C-AFM	–0.248	–0.424	–0.414
G-AFM	–0.228	–0.375	–0.408

a The FM ordering is set as the zero of energy.

**Figure 6:**
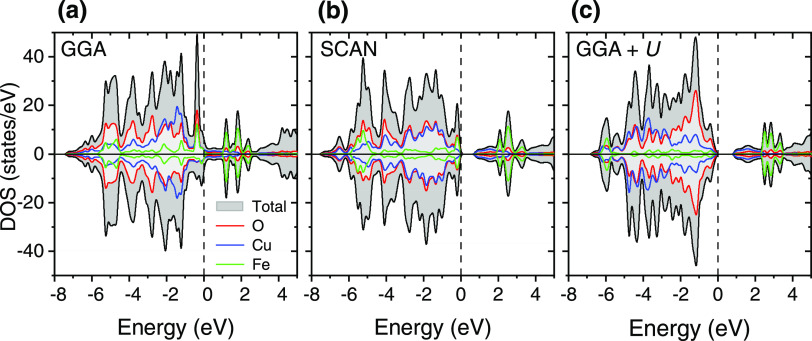
Calculated total and atom-projected density of states (DOS) for the ground-state C-magnetic structure of the idealized YSr_2_Cu_2_FeO_7_: (a) GGA functional, (b) SCAN functional, and (c) GGA + *U* method. The Fermi level is set as the zero of energy. Up-spin (or majority) and down-spin (or minority) contributions are shown. Color code: total black, Cu contribution in blue, Fe contribution in green, and O contribution in red. DOS units refer to the calculated cell.

Independently on the utilized functional, the optimization of the crystal structure of the idealized YSr_2_Cu_2_FeO_7_ oxide results in lattice parameters in reasonable agreement with the experimental values of YSr_2_Cu_2_FeO_7.08_ with errors below 2.1% (Table 2 and Figure S1). The GGA and GGA + *U* tend to overestimate the lattice parameters and TM–O distances, while the SCAN functional underestimates them. For the Fe environment, all of the approximations give a satisfactory description of the average bond distances (errors below 0.7%), and even of the corrugation of the [FeO] layers, the SCAN results being the closest to the experimental values. However, the Cu–O square pyramidal environment is more elongated than the one experimentally observed (*d* axial/*d* equatorial, *d* Cu–O1/d Cu–O2 in Table 2). Since the hybridization of the Cu-apical oxygen plays a significant role in the electronic structure of YBCO-related oxides,^51^ it is worth noting that the deviations in the Cu–O1 bond lengths are of 5.5% (GGA), 3.6% (GGA + *U*), and 1.7% (SCAN). According to the major deviation in the Cu–O1 bond distance, the GGA fails to reproduce the semiconducting behavior of YSr_2_Cu_2_FeO_7.08_ (calculated DOS in Figure 6a) in clear disagreement with the experimental observations (Figure 3b).

While the GGA incorrectly predicts the metallic behavior for the ground state of idealized YSr_2_Cu_2_FeO_7_, a band gap of 0.85 eV opens in the GGA + *U* (*U*-Cu and *U*-Fe = 4 eV), and noteworthy, a near band gap of 0.63 eV is obtained using the SCAN functional (Figure 6). Compared to the YSr_2_Cu_2_FeO_8_ phase, in both SCAN and GGA + *U*, the effective charge of the TMs decreases (Table 3), and the TM–O distances increase (Table 2), in agreement with the lower formal oxidation states of Fe^3+^ and Cu^2+^ in YSr_2_Cu_2_FeO_7_. The effective charge on oxygen ions increases, approaching the oxide ion O^2–^. SCAN and GGA + *U* methods predict nearly the same calculated magnetic moments for TM ions: μ(Cu) = 0.5 μ_B_ per atom and μ(Fe) ∼ 4 μ_B_ per atom. The magnetic moment of the Cu ions is in good agreement with the experimental values reported in similar Cu^2+^ compounds.^52,53^ The magnetic moment on Fe ions suggests a high-spin Fe^3+^ configuration (*e*^2^*t*_2_^3^, magnetic moment of the free ion μ_0_ = 5.0 μ_B_). It is worth to note that magnetic moment values between 3 and 4 μ_B_ per atom have been experimentally observed in other complex Fe^3+^-tetrahedral oxides.^54,55^ On the other hand, the Fe–O and Cu–O covalencies bring an appreciable spin moment of ∼0.2 μ_B_ in the shared O1 site. As observed in the atom-projected density of states (Figure 6c), the *U* term shifts the TM-3*d* states to lower energies; yet, there is a good hybridization of TM-3*d* to O-2*p* states (see band at −6 eV). In summary, for the insulating compound, SCAN offers very similar results to the GGA + *U* in terms of band-gap and magnetic moments, although there are clear differences in the shape and nature of the states at the Fermi level. To further analyze the appropriateness of the SCAN vs. the GGA + *U* method, in a first approximation, the calculated DOS could be qualitatively compared with experimental photoelectron spectroscopy (PES). It is however important to point out that such comparison neglects the excitation aspects, which can be taken into account by quasi-particle calculations in the GW approach.^56^

For the sake of completeness, the relation between the electronic properties and the magnetic structure of YSr_2_Cu_2_FeO_7_ is analyzed within the SCAN and GGA + *U* methods (Figure S2). Once more, both DFT approximations yield the same results in terms of the insulating/metallic behavior. The calculated DOS for the G magnetic structure, with in-plane Cu/Fe AFR interactions, corresponds to an insulating compound with the same band gap of the C-magnetic structure (0.63 eV in SCAN, 0.85 eV in GGA+ *U*). The FR and A magnetic structures, with in-plane Cu/Fe FR interactions, generate metallic properties. These results reveal that, for the idealized YSr_2_Cu_2_FeO_7_, the insulating character couples to the in-plane AFM Cu/Fe ordering.

#### 3.2.3 On the Metal-to-Insulating Transition in the YSr_2_Cu_2_FeO_8−δ_ (0 < δ < 1) Family

**Figure 7:**
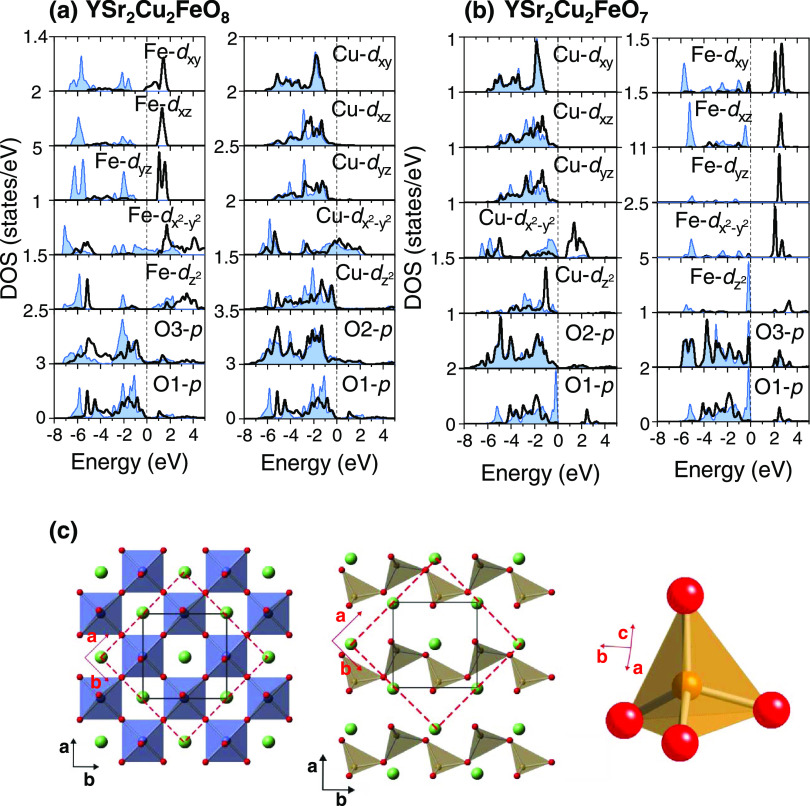
Orbital-projected density of states within the SCAN functional for (a) idealized YSr_2_Cu_2_FeO_8_ showing the Fe-3*d*, O3-2*p* and O1-2*p* and Cu-3*d*, O2-2*p*, and O1-2*p* orbitals, and (b) idealized YSr_2_Cu_2_FeO_7_ showing the Cu-3*d*, O2-2*p* and O1-2*p* and Fe-3*d*, O3-2*p,* and O1-2*p* orbital contribution. Color code: up-spin (or majority) in blue and down-spin (or minority) in black. (c) Crystallographic cell used for the orbital-projected DOS of YSr_2_Cu_2_FeO_7_ (in red) and its relation to the to the crystal setting in the *Ima*2 S.G. (in black).

The above results highlight that the SCAN functional reproduces the metal and the insulating character of YSr_2_Cu_2_FeO_8_ and YSr_2_Cu_2_FeO_7_ compounds, respectively. The orbital-projected DOS (Figure 7) adds more insights into the comprehensive interpretation of the chemical bonding and the evolution of the electronic properties as a function of the δ value. Starting with the metallic YSr_2_Cu_2_FeO_8_, in the Fe octahedra, the hybridization of O3(2*p*)–Fe(3*d_x^2^–y^2^_*) and O1(2p)–Fe(3*d_z_^2^*) produces the lower energy band σ-O(*p*)–Fe(*e*_g_) and the upper energy band σ*-O(*p*)–Fe(*e*_g_). As observed in Figure 7a, the occupation of the bonding sigma band occurs in both spin channels, with the Fermi level crossing the 3*d_x^2^–y_*_^*2*^_ majority spin orbital. The Π O(2*p*)–Fe(3*t*_2g_) bands are fully occupied in the up-spin channel, but also show some occupancy in the down-spin channel. As deduced from the calculated magnetic moments, the 3*d*-Fe orbitals filling does not correspond to either LS-Fe^4+^ (*t*_2g_^4^*e*_g_^0^) or HS-Fe^4+^ (*t*_2g_^3^*e*_g_^1^), but to an itinerant character where every 3*d* orbital contributes to the magnetic moment, in agreement with the metallic properties of the oxide. For the Cu-3*d* states, the crystal field splitting of the Cu–O square pyramidal environment lifts the degeneracy of the Cu-*e_g_* orbitals to the lower-lying 3*d_z_*_^*2*^_ and higher-lying 3*d_x^2^–y_*_^2^_ orbitals. The strong hybridization O2(2*p*)–Cu(3*d_x^2^–y_*_^*2*^_) produces a wide band that is partially filled in both spin channels, yielding a low net spin of 0.3 μ_B_. The Fermi level lies in the band constructed mainly from the Cu-3*d_x^2^–y^2^_* and O2-2*p* orbitals and in a minor extent in the band constructed from Fe-3*d_x^2^–y_*_^*2*^_ and O3-2*p* orbitals. Therefore, these bands have the largest implication in the electronic properties of the YSr_2_Cu_2_FeO_8−δ_ family.

Upon vacancies incorporation in YSr_2_Cu_2_FeO_8_ (Figure 7a) to form YSr_2_Cu_2_FeO_7_ (Figure 7b), according to the Fermi-level displacement, electron filling occurs in the Cu-3*d_x^2^_*_*–y*__^2^_ derived band. Since in YSr_2_Cu_2_FeO_7_ the Cu ions have the nominal valence of 2+, and hence a nominal *d*^9^ state, there are fully occupied *t*_2__g_ orbitals and *d_z^2^_* orbitals, while the *d*_*x^2^–y^2^*_ orbital remains half-filled yielding a low net spin of 0.5 μ_B_. The energy gap opens between the occupied and the unoccupied Cu-3*d_x^2^–y^2^_* orbitals. On the other hand, the analysis of the Fe-3*d* states upon electron doping of YSr_2_Cu_2_FeO_8_ is more complicated, since the coordination around the Fe atoms changes from octahedral in YSr_2_Cu_2_FeO_8_ to tetrahedral in YSr_2_Cu_2_FeO_7_. According to crystal field theory, in the tetrahedral field, the Fe-3*d* orbitals split into two manifolds, a lower one with two levels and an upper one with three levels (these would be of *e* and *t_2_* character respectively, if the local symmetry were perfectly tetrahedral). This is not observed in Figure 7b due to the orientation of the Fe tetrahedra relative to the crystallographic cell axes utilized for the orbital-projected DOS (Figure 7c). Nevertheless, for all of the Fe-3*d* states, the up-spin channel is fully occupied and the down-spin channel is almost empty, the energy separation between up and down-spin channels being rather large. This electron density localization leads to the aforementioned high-spin Fe^3+^ configuration.

In summary, the results of the SCAN methodology indicate that in the YSr_2_Cu_2_FeO_8−δ_ family, the lowering of the oxygen content drives the metal-to-insulating transition by electron doping. A reduction in the oxygen content (higher δ value) results in a decrease in the nominal valence of Cu/Fe ions, and hence in the filling of the Cu/Fe-3*d_x^2^–_*_*y^2^*_ states. This picture is fully consistent with the well-documented importance of filling control in the electronic structure of high *T*_c_ cuprates, a typical example being the insulating to metal transition in La_2–*x*_Sr*_x_*CuO_4_ induced by hole doping (*x* = 0 metallic).^18,57^

Remarkably, the SCAN functional offers a feasible interpretation of the metal-to-insulating transition in the YSr_2_Cu_2_FeO_8−δ_ family, when looking at the idealized compounds with δ = 0 and 1. However, in addition to distinct oxygen contents (δ values), the synthesized compounds YSr_2_Cu_2_FeO_7.86_ (δ = 0.14) and YSr_2_Cu_2_FeO_7.08_ (δ = 0.92) show antisite disorder, with a Cu/Fe mixing close to 15% (see Table 1 and ref (24)). As noted by different authors,^22−24,58,59^ the mutual substitution of Cu and Fe depends on the annealing history/oxygen content. The simulation of the random distribution of the antisite defects that occurs in the real materials requires the utilization of the special quasi-random structures (SQS) approach,^60^ which is out of the scope of this work. The antisite disorder could perturb the AFM in-plane ordering that, as discussed above, is critically linked to the insulating behavior of the investigated compounds with the lowest oxygen contents (δ ∼ 0). It is foreseeable that the concentration and location of both oxygen vacancies and antisite defects drastically influence the magnetic and electronic properties of the YSr_2_Cu_2_FeO_8−δ_ family. This is in agreement with the observed dependence between the property and the synthetic history of the materials.

## 4 Conclusions

The precise modeling of the ground-state properties in complex oxides that contain more than one type of TM ions remains challenging, especially with the irruption of correlated oxides as functional materials. In this work, we have examined the capabilities of different DFT methodologies (GGA, GGA + *U*, SCAN) to model the crystal structure and the electronic and magnetic properties of YSr_2_Cu_2_FeO_8−δ_ compounds in which the oxygen content in the FeO_2−δ_ layers drives the average oxidation states of both Fe and Cu cations, thereby determining the electrical properties. We have found that the SCAN and GGA methodologies are valid to simulate the metallic properties of the oxide with the highest oxygen content of the family (δ = 0, idealized-YSr_2_Cu_2_FeO_8_) and the itinerant character of its bonding electrons. Introducing the *U* term (GGA + *U*) results in excessive electron localization and large magnetic moments that deviate from the experimental observations, therefore discarding this methodology for property prediction in the metallic phases of the YSr_2_Cu_2_FeO_8−δ_ system. Contrariwise, the insulating character of the compound with the lowest oxygen content (δ = 1, idealized-YSr_2_Cu_2_FeO_7_) is well captured when electron correlations are treated by the *U* parameter using the GGA + *U* approximation. Importantly, the results point out that the SCAN functional also offers a good platform to investigate these insulating compounds. These results render the SCAN functional as the only one well suited, among the utilized methodologies, to investigate the basic electronic properties across the YSr_2_Cu_2_FeO_8−δ_ series. It should be noted that, together with the lack of an adjustable *U* parameter, other benefits of the SCAN functional are the affordable computational cost and the transferability of results among different works. Yet, a severe limiting factor for the DFT investigation of the YSr_2_Cu_2_FeO_8−δ_ family resides in the need of complex crystallographic models to take into account the antisite Cu/Fe disorder and the fractional occupancies of the O3 site that occur in the synthesized materials.

## Supporting Information Available

Calculated lattice parameters and density of states for the A-AFM, G-AFM, and FM magnetic structures of YSr_2_Cu_2_FeO_7_ (PDF)
