# Supplementary material for: Metal-to-Insulating Transition in the Perovskite System YSr2Cu2FeO8−δ (0 < δ < 1) Modeled by DFT Methods
Source: Inorg Chem. 2023 Feb 14;62(8):3445–56. doi: 10.1021/acs.inorgchem.2c03475 (PMC9976288; doi:10.1021/acs.inorgchem.2c03475)
Supplement: Supplementary file 1 — ic2c03475_si_001.pdf [file ic2c03475_si_001.pdf]

Supporting Information

Metal to insulating transition in the perovskite system $YSr_2Cu_2FeO_{8-\delta}$ ($0 < \delta < 1$) modeled by DFT methods

Marianela Gómez-Toledo[1], Sara A. López-Paz[1, 2], Susana García-Martín[1] and M. Elena Arroyo-de Dompablo[1]*

1. Departamento de Química Inorgánica, Universidad Complutense de Madrid, 28040 Madrid, Spain.

2. Department of Quantum Matter Physics, University of Geneva, CH-1211, Geneva, Switzerland

Figure S1. Calculated lattice parameters for the C-AFM, G-AFM, A-AFM and FM magnetic structures of $YSr_2Cu_2FeO_7$

Figure S2. Calculated Density of States for the A-AFM, G-AFM and FM magnetic structures of $YSr_2Cu_2FeO_7$

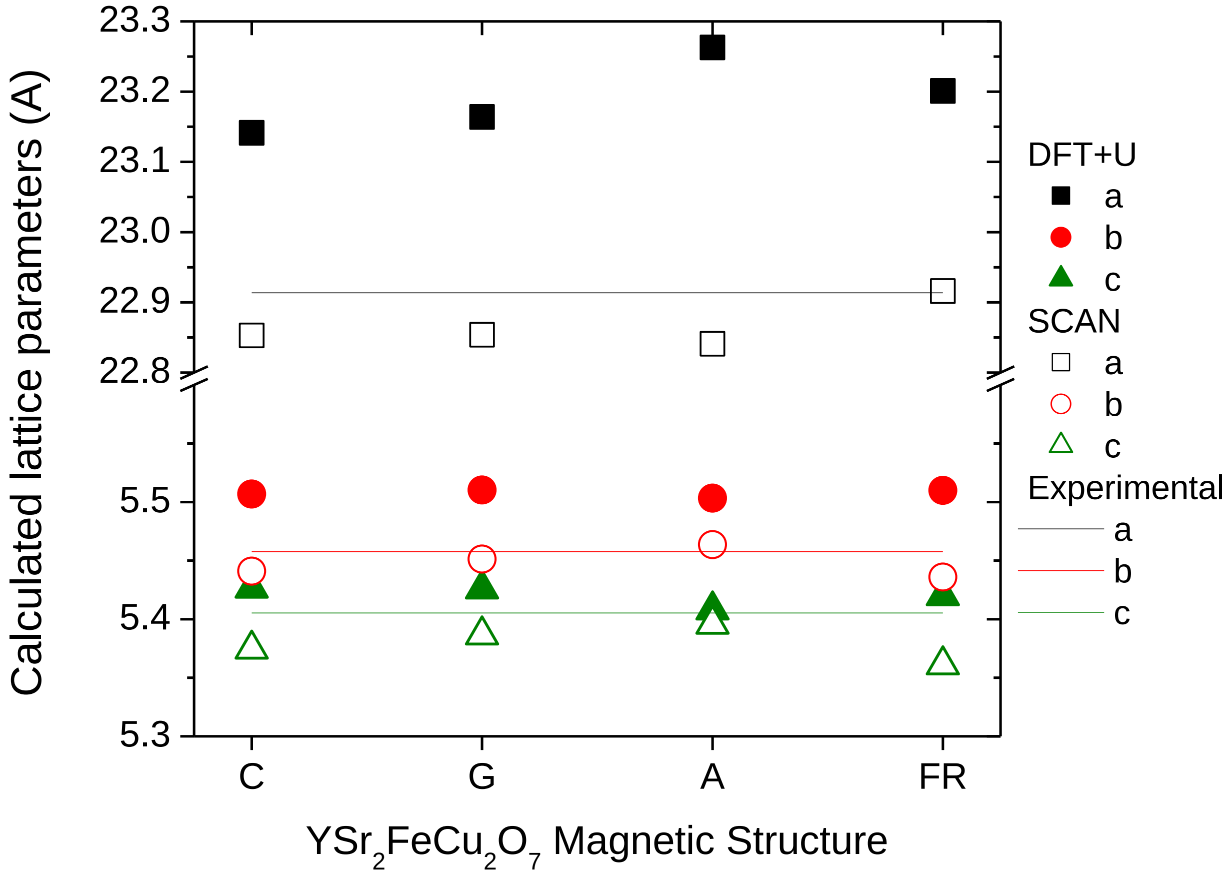

Figure S1. Calculated lattice parameters for the idealized $YSr_2Cu_2FeO_7$ in the C, G, A and FR magnetic structures (S.G. *I*ma2). The experimental data of $YSr_2Cu_2FeO_{7.08}$ (Table I in main text) are included for comparison.

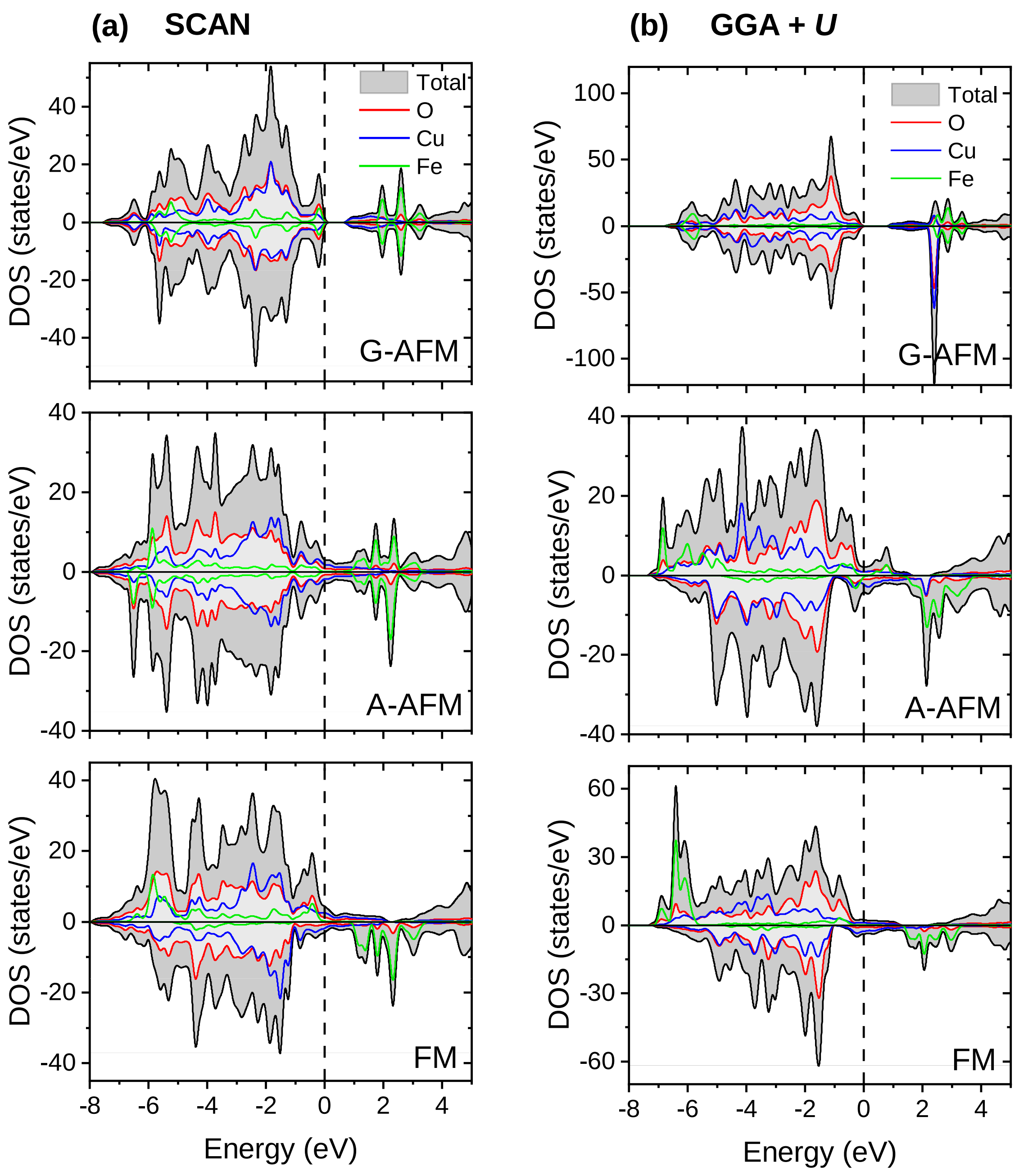

Figure S2. Calculated total and atom-projected density of states for idealized $YSr_2Cu_2FeO_7$ in the G, A and FR magnetic structures. (a) SCAN functional and (b) GGA+*U* method. The Fermi level is set as the zero of energy. Up spin (or majority) and down spin (or minority) contributions are shown. Color code: total grey, Cu contribution in blue, Fe contribution in green, O contribution in red. DOS units refer to the calculated cell.
